# Fluid challenge and left ventricular myocardial performance index assessed by transthoracic echocardiography with tissue Doppler imaging in critically ill patients with regular rhythm

**DOI:** 10.3389/fmed.2026.1885708

**Published:** 2026-06-24

**Authors:** Mircea Tamas Talpoș, Halit Ozel, Basil Khaled Mohamed Sallam, Dimitrios Velissaris, Rachid Attou, Charalampos Pierrakos

**Affiliations:** 1 Department of Intensive Care, Brugmann University Hospital, Université Libre de Bruxelles, Brussels, Belgium; 2 Department of Internal Medicine, University Hospital of Patras, Patras, Greece

**Keywords:** diastolic dysfunction, fluid challenge, hemodynamic monitoring, myocardial performance index, noninvasive assessment, preload, transthoracic echocardiography, transthoracic ultrasound

## Abstract

**Background:**

Fluid challenge may increase cardiac index but can also compromise diastolic function. The myocardial performance index, derived from tissue Doppler imaging, integrates systolic and diastolic performance into a single measure that may reflect the balance between the benefits and risks of fluid challenge. We hypothesized that patients with an increase in cardiac index after fluid challenge would demonstrate a reduced myocardial performance index, indicating improved global function, whereas those without an increase in cardiac index would display stable or higher values, suggesting limited or adverse effects.

**Methods:**

This secondary analysis of a prospective observational study was conducted in the intensive care unit of Brugmann Hospital, Belgium, between October 2020 and December 2023. Patients with regular rhythm who received a fluid challenge of 4 mL/kg were included. Fluid responsiveness was defined as an increase in cardiac index of at least 15%, and myocardial performance index values below 0.42 were considered normal. The primary endpoint was the change in myocardial performance index during the fluid challenge.

**Results:**

Of 59 patients, 24 (41%) were fluid responders. Among the responders, myocardial performance index decreased significantly after fluid challenge (from 0.48 [0.43–0.58] to 0.42 [0.33–0.54]; *p* < 0.01). Among non-responders, no significant change was observed (from 0.49 [0.41–0.68] to 0.48 [0.41–0.81]; *p* = 0.28). Normalization of the myocardial performance index after fluid challenge was significantly associated with fluid responsiveness (odds ratio, 3.37; 95% confidence interval, 1.12–10.78, *p* = 0.01).

**Conclusion:**

In critically ill patients with regular cardiac rhythm, the myocardial performance index decreased after fluid challenge in responders but not in non-responders, suggesting improved global myocardial function. These findings support the potential role of the myocardial performance index as a dynamic marker of cardiac response to fluid challenge.

## Introduction

Fluid challenge (FC) is a key intervention for critically ill patients, involving rapid intravenous fluid administration with simultaneous hemodynamic monitoring (1). However, assessing its hemodynamic effects is challenging and lacks standardization (2). Measurement of the cardiac index (CI) before and after FC is considered the most reliable approach for evaluating hemodynamic changes because parameters derived from arterial pressure may provide misleading estimates of fluid responsiveness (3, 4). Transthoracic echocardiography (TTE), a minimally invasive Doppler-based method for measuring CI, is widely used in clinical practice to evaluate the effects of FC. Nevertheless, its accuracy may be limited by assumptions about flow profiles and imaging angles, which can hinder the detection of subtle CI changes (5, 6). In addition, focusing exclusively on CI during FC may lead to overlook of important alterations in diastolic function, potentially offsetting any benefits (7, 8). Therefore, improving TTE-based monitoring during FC is a critical research priority to achieve a more comprehensive understanding of the hemodynamic consequences of bedside fluid administration.

The myocardial performance index (MPI), first described by Tei, is a transthoracic echocardiographic parameter that integrates both systolic and diastolic functions of the left ventricle (9, 10). MPI is calculated as the ratio of isovolumic contraction and relaxation times to ejection time (ET). Because contraction and relaxation are energy-dependent processes, dysfunction prolongs the isovolumic phases, thereby increasing MPI and reducing global left ventricular efficiency (11). FC influences cardiac performance in different ways: preload enhancement can improve systolic function through the Frank–Starling mechanism (7), whereas diastolic function may be impaired (12). Therefore, evaluating MPI during FC is of particular interest as it can capture the net impact of preload expansion on overall ventricular performance. Tissue Doppler imaging-derived MPI (TDI-MPI), obtained from mitral annulus velocity curves, enables precise delineation of systolic and diastolic phases within a single cardiac cycle (13). By detecting subtle abnormalities in contractility or relaxation that may be overlooked by conventional indices such as CI or ejection fraction (EF) (14, 15), TDI-MPI may improve the assessment of FC, particularly in borderline or heterogeneous hemodynamic responses where reliance on CI thresholds alone is inconclusive (16).

This study aimed to evaluate the physiological response of TDI-MPI during FC in critically ill patients. We hypothesized that an increase in CI after FC would correlate with improved overall cardiac function, primarily reflecting enhanced systolic performance without deterioration of diastolic function, thereby resulting in a net decrease in TDI-MPI. Conversely, in patients without an increase in CI after FC, MPI would remain unchanged or increase, indicating inadequate systolic compensation and worsening diastolic function.

## Methods

### Design and setting

This study is a secondary analysis of a prospective observational trial conducted in the 21-bed intensive care unit (ICU) of Brugmann University Hospital, Brussels, Belgium. The original protocol was registered before patient enrollment in the ISRCTN registry (ISRCTN58464956). Patients who underwent FC between October 2020 and December 2023 were eligible, and each participant was evaluated only once. The original study investigated changes in the central venous-to-arterial carbon dioxide tension difference (P_va_CO_2_) and capillary refill time during FC in critically ill patients with suspected hypovolemia. For the present analysis, we focused on patients who underwent echocardiographic evaluation before and after FC as part of routine practice. Some of these patients were also included in the original study, whereas others had been excluded because they did not present with elevated baseline P_va_CO_2_.

The present study was approved by the local ethics committee (CE2021/29). Informed consent was obtained from all enrolled patients or, when not feasible, from the patient’s next of kin, including for those excluded from the primary study. This study complied with the Declaration of Helsinki (17) on research involving humans and followed Good Clinical Practice guidelines for data security and patient confidentiality. Administration of FC was at the discretion of the attending physician.

### Inclusion and exclusion criteria

Critically ill adult patients (age > 18 years) who received FC > 6 h after ICU admission and were evaluated using TTE were eligible. The exclusion criteria were as follows: interventions within 1 h before FC such as an increase in inotrope dosage or initiation of mechanical ventilation; veno-arterial extracorporeal membrane oxygenation support; FC performed for acute bleeding; life-threatening hemodynamic instability; irregular rhythm, which may cause fluctuations in cardiac cycle intervals and reduce the reliability of pre- and post-FC comparisons; and inability to determine CI and TDI-MPI before and after FC.

### FC technique

All patients underwent the standardized fluid challenge protocol described previously (18). Each patient received crystalloids (Plasma-Lyte A®, Baxter Healthcare, Deerfield, IL, United States) at a dose of 4 mL/kg of actual body weight, administered at 1.2 L/h via an infusion pump. For workflow efficiency, the prescribed volume was rounded to the nearest 50 mL increment, ranging from 250 to 500 mL (i.e., 250, 300, 350, 400, 450, or 500 mL). No additional interventions were permitted during FC. The decision to perform FC and its clinical objective were determined by the attending physician. However, for the purposes of this study, fluid responsiveness was defined as an increase in CI ≥ 15%; changes in arterial pressure were not used to define a positive hemodynamic response.

### Data and sample collection

Demographic data, concurrent treatments (mechanical ventilation and inotropic agents), and laboratory results were recorded for each patient. Disease severity at admission was assessed using the Simplified Acute Physiology Score (SAPS) 3. Data collection followed the CODEFIRE consensus recommendations for standardized reporting of FC protocols in critically ill patients (19).

### TTE examination

TTE was performed by physicians trained in critical care ultrasound using the ARIETTA™ 650 Deep Insight Ultrasound System (FUJIFILM Healthcare Corporation, The Netherlands). Examinations were conducted with patients in the supine position, avoiding significant mobilization, by physicians experienced in cardiac ultrasound in critically ill patients (MTT and CP). Continuous cardiac rhythm monitoring was maintained during each examination. Left heart function was assessed using parasternal, apical, and subcostal windows. All images and videos were digitally stored and analyzed offline by a single investigator (MTT). Echocardiographic measurements were based on the median of three consecutive cardiac cycles. For CI, a change of ≥15% was considered the least significant detectable difference (5).

Stroke volume (SV) was calculated according to the continuity principle, which assumes a constant flow through the heart and vascular system, as the product of the left ventricular outflow tract (LVOT) area (derived from the LVOT diameter) and the velocity–time integral measured by Doppler echocardiography. Assuming circular geometry, LVOT diameter was measured just below the aortic valve in the parasternal long-axis view. Cardiac output was calculated as the product of SV and heart rate, and CI was derived by normalizing the cardiac output to body surface area.

Pulsed-wave Doppler was used to evaluate blood velocities. The early diastolic wave (E), reflecting rapid left ventricular filling, and the late diastolic wave (A), reflecting atrial contraction, were measured, and the E/A ratio was calculated. TDI, combined with electrocardiographic readings, was used to assess the motion of the lateral mitral annulus. The diastolic waves E′ and A′, along with the systolic wave S′, were measured. The isovolumetric contraction time (IVCT), isovolumetric relaxation time (IVRT), and ET were obtained from the TDI trace. TDI-MPI was calculated as the ratio of the sum of the IVCT and IVRT to ET (Supplementary Figure S1).

### Definitions

Sepsis and septic shock were defined according to the Sepsis-3 definition (20). Cardiogenic shock was defined as a syndrome in which a low cardiac index (≤2.2 L/min/m^2^) and EF < 50% result in inadequate tissue perfusion associated with a systolic blood pressure below 90 mmHg for more than 30 min or the need for inotropes, vasopressors, to maintain adequate blood pressure (21). Patients were considered fluid responders if their CI increased by ≥15% (5, 22). Abnormal TDI-MPI was defined as ≥0.42 (23). Diastolic dysfunction was defined as E/e′ > 8 (12). E/e′ was selected instead of e′ alone because e′ mainly reflects myocardial relaxation, whereas E/e′ better estimates filling pressures (24), a clinically relevant safety concern during FC.

### Outcomes

The primary outcome of this study was TDI-MPI. The secondary outcomes were ET, IVRT, and IVCT. In addition, 28-day ICU survival probability and discharge rates were assessed.

### Analysis plan

Because this was an exploratory secondary analysis, formal sample size calculation was not performed; all eligible patients from the primary study were included. Descriptive statistics were generated, with categorical data reported as frequencies (%) and continuous variables as medians (IQR). Absolute (*Δ* = Post–Pre) and relative ([Post–Pre] / Pre × 100) changes were calculated. Between-group differences are expressed as standardized mean differences (SMD), using conventional thresholds of 0.2, 0.5, and 0.8 to indicate small, medium, and large effects, respectively (25).

Changes in TDI-MPI and its components (IVCT, IVRT, and ET) before and after FC were tested with paired Wilcoxon signed-rank tests, stratified by fluid responsiveness. Patients were classified as “MPI normalizers” if MPI improved from abnormal (≥0.42) to normal (<0.42) after FC or remained normal; all others were “non-normalizers.” Logistic regression (univariate due to sample size constraints) was used to assess the association between MPI normalization and fluid responsiveness, with odds ratios and 95% confidence intervals.

Patient outcomes were modeled using multistate competing risk proportional hazards (ICU discharge and mortality as competing events, persistent ICU stay as the reference, and censoring at 28 days). In parallel, Bayesian Cox survival models (brms package) were applied to estimate posterior hazard ratios and certainty levels (≥95% high, 80–94% moderate, <80% inconclusive) (26).

Sensitivity analyses evaluated whether MPI and its components (IVCT, IVRT, and ET) responded differently to FC according to baseline diastolic function, using linear mixed-effects models with interaction terms (time × diastolic dysfunction). Interaction plots illustrated pre- and post-FC trajectories.

All statistical analyses were conducted using R software (version 4.4.1) via the RStudio platform.^1^ Statistical significance was set at a *p*-value of <0.05. TDI-MPI was considered the sole primary outcome. Analyses of IVCT, IVRT, and ET were secondary and exploratory; therefore, no formal multiplicity correction was applied.

## Results

### Study patients

Among the 105 evaluated patients, 59 were enrolled in this study (Supplementary Figure S2), most of whom were assessed on the first day of ICU admission (1 [0–3] days). Among these, 24 patients (41%) were fluid responders. Pre- and post-FC hemodynamic changes are shown in Supplementary Table S1. There were no significant differences in severity between responders and non-responders. Diastolic dysfunction and hyperlactatemia were more common among non-responders (Table 1). Hemodynamic and echocardiographic parameters, including TDI-MPI, showed minimal variation between the groups (Supplementary Table S2). Non-responders also had a higher central venous pressure (CVP) and heart rates than responders.

**Table 1:** Characteristics of patients by fluid challenge response and diastolic dysfunction.

Patients characteristics	FC responders			FC non-responders			SMD
	Total population	Diast. Dfc.	No Diast Dfc	Total population	Diast. Dfc.	No Diast Dfc	
No of patients	24	10	14	35	17	18	
Age (years)	66 (54–78)	65 (56–70)	68 (55–79)	69 (52–75)	71 (55–75)	67 (51–75)	0.029
APACHE II	15 (13–20)	14 (12–17)	16 (14–22)	16 (11–23)	17 (10–24)	14 (11–24)	0.139
SAPS III	62 (46–67)	52 (48–75)	65 (45–66)	59 (49–72)	62 (45–76)	57 (50–64)	0.218
Female	10 (42)	6 (60)	4 (28)	17 (48)	9 (53)	8 (44)	0.137
Height (cm)	169 (165–178)	167 (158–173)	175 (165–178)	165 (160–176)	162 (160–174)	167 (160–180)	0.158
Weight (kg)	73 (65–82)	73 (63–81)	76 (66–85)	78 (70–90)	79(70–90)	76(70–85)	0.393
Volume of fluid (ml)	300 (270–342)	300 (262–337)	300 (280–335)	300 (300–400)	300 (300–400)	300 (300–350)	0.283
Normalized fluid volume to actual body weight (mL/kg)	4.1 (4.0–4.4)	4.2 (4.0–4.6)	4.0 (3.9–4.4)	4.1 (4.0–4.3)	4.1 (4.0–4.4)	4.0 (3.9–4.2)	0.297
Duration of FC	15 (13–17)	15 (14–17)	15 (13–17)	15 (15–20)	15 (15–20)	15 (15–17)	0.283
Comorbidities							
Hypertension	14 (58)	7 (70)	7 (50)	21 (60)	9 (53)	12 (66)	0.017
Diabetes	12 (50)	5 (50)	7(50)	17 (48)	9 (53)	8 (44)	0.071
Ischemic cardiomyopathy	4 (17)	3 (30)	1 (7)	7 (20)	2 (12)	5 (31)	0.061
Heart failure with reduced EF	2 (8)	2 (20)	0 (0)	1 (3)	1 (6)	0 (0)	0.247
Reason for ICU admission							
Acute respiratory failure	4 (16)	1 (10)	3 (21)	5 (14)	2 (12)	3 (17)	0.084
Sepsis	7 (29)	3 (30)	4 (29)	16 (46)	7 (41)	9 (50)	0.313
Cardiovascular disease	0 (0)	0 (0)	0 (0)	2 (6)	2 (12)	0 (0)	0.343
Pancreatitis	0 (0)	0 (0)	0 (0)	1 (3)	1 (6)	0(0)	0.091
Neurological disease	1(4)	0 (0)	1 (7)	4 (11)	3 (18)	1 (5)	0.198
Other	11 (46)	6 (60)	7 (50)	7 (20)	2 (12)	5 (27)	0.369
Clinical situation at the time of FC							
Sepsis	14 (58)	6 (60)	8 (57)	24 (68)	11 (64)	13 (72)	0.159
Shock							
Septic	5 (21)	3 (30)	2 (14)	14 (40)	5 (29)	8 (44)	0.696
Hypovolemic	5 (21)	4 (40)	1 (7)	2 (6)	0 (0)	2 (11)	0.868
Cardiogenic	0 (0)	0 (0)	0 (0)	1(3)	1 (6)	0 (0)	0.346
Invasive ventilation	9 (37)	6 (60)	3 (21)	16 (45)	11 (65)	5 (28)	0.131
Reason for FC							
Hypotension	11 (45)	6 (60)	5 (35)	12 (34)	6 (35)	6 (33)	0.273
Hyperlactatemia	7 (29)	3 (30)	4 (28)	18 (51)	8 (47)	10 (55)	0.429
Oliguria or other signs of hypo-perfusion	5 (21)	1 (10)	4 (28)	5 (14)	3 (18)	2 (11)	0.191

APACHE II, Acute Physiology and Chronic Health Evaluation II score; EF, Ejection Fraction; FC, Fluid Challenge; ICU, Intensive Care Unit; SAPS III, Simplified Acute Physiology Score III.

Values are presented as median (interquartile range) or number (%). Standardized mean differences (SMD) compare the total populations of responders and non-responders. Fluid responders were defined as patients with an increase in cardiac index of ≥15% after fluid challenge. Diastolic dysfunction was defined by E/e′ > 8.

### Changes in TDI-MPI according to fluid response

**Figure 1:**
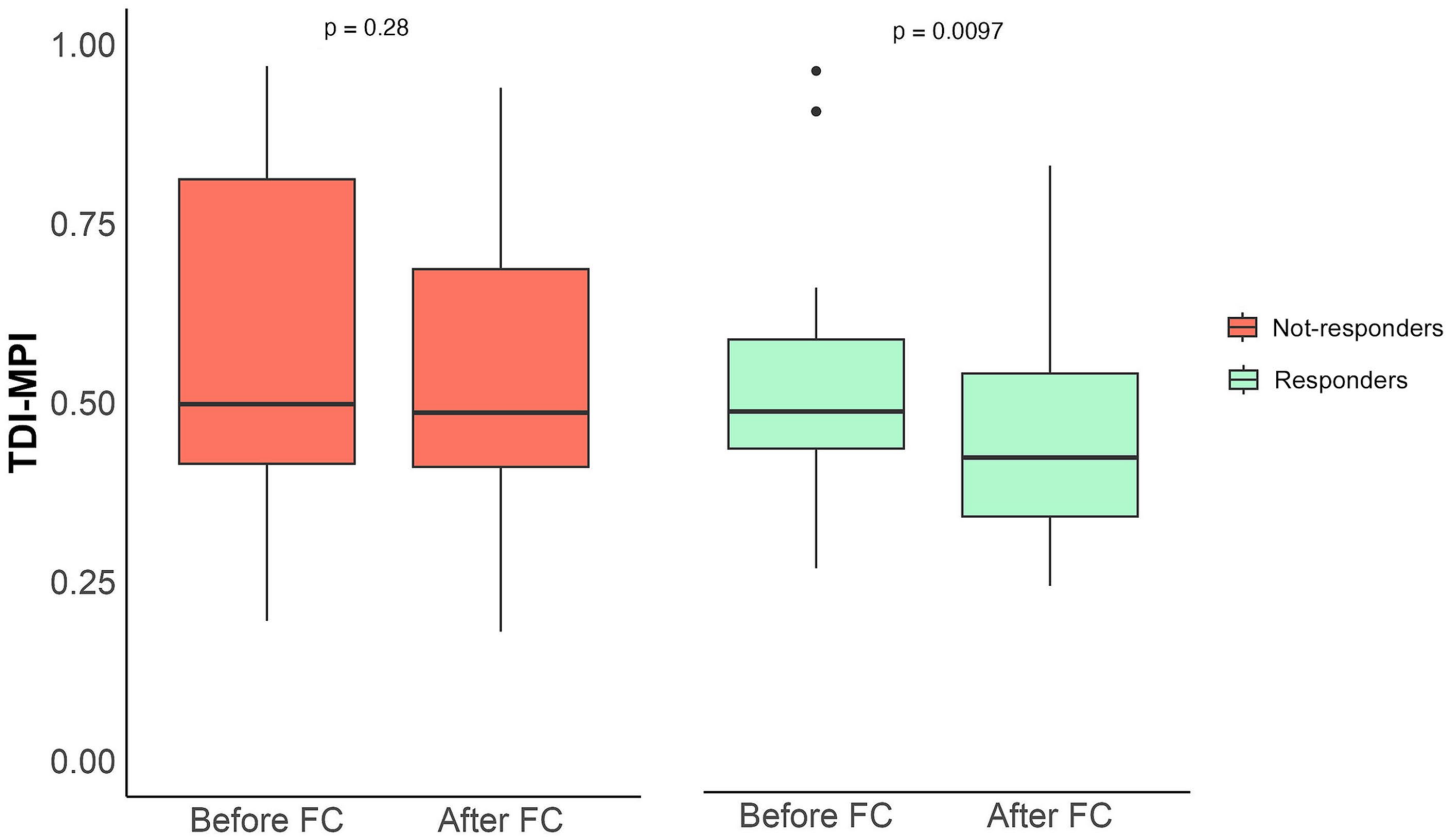
Myocardial performance index (MPI) before and after fluid challenge in responders and non-responders. Boxplots showing tissue Doppler imaging-derived MPI values before and after fluid challenge in fluid responders (green) and non-responders (red). The boxes represent the interquartile range, the horizontal line within each box indicates the median, and the whiskers show the full range excluding outliers. Data were analyzed using the paired Wilcoxon signed-rank test. Corresponding *p*-values are displayed above each comparison.

TDI-MPI decreased from 0.48 [0.43–0.58] before FC to 0.42 [0.33–0.54] after FC in fluid responders (*p* < 0.01), whereas no significant change was observed in non-responders (0.49 [0.41–0.68] to 0.48 [0.41–0.68], *p* = 0.28) (Figure 1). MPI normalization was significantly associated with fluid responsiveness, particularly among patients with diastolic dysfunction (Supplementary Figure S5).

### Changes in the TDI-MPI components according to fluid response

**Figure 2:**
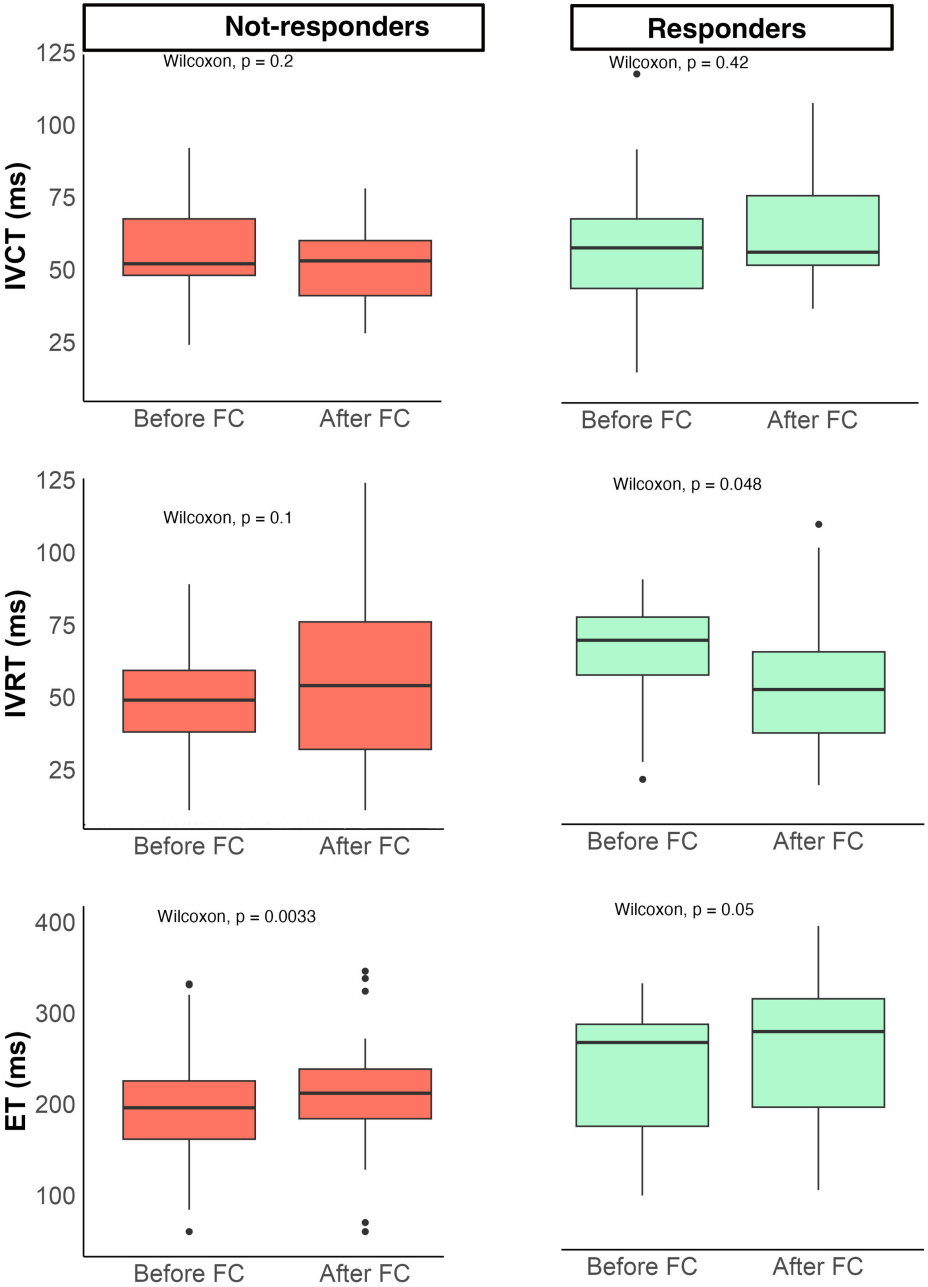
Components of myocardial performance index before and after fluid challenge in responders and non-responders. Boxplots showing tissue Doppler imaging-derived components of the myocardial performance index before and after fluid challenge in responders (green) and non-responders (red). Panels display isovolumetric contraction time (IVCT, top), isovolumetric relaxation time (IVRT, middle), and ejection time (ET, bottom). The boxes represent interquartile ranges, the horizontal lines indicate medians, and whiskers show ranges excluding outliers. Statistical comparisons were performed using paired Wilcoxon signed-rank tests, with corresponding p-values displayed above each panel.

Fluid responders showed a greater decrease in IVRT than in IVCT after FC, whereas non-responders demonstrated negligible changes in either parameter. ET increased in both groups after FC compared with baseline values (Figure 2).

### Changes in TDI-MPI and its components after FC according to diastolic dysfunction

TDI-MPI did not differ between patients with and without diastolic dysfunction (0.47 [0.41–0.63] vs. 0.49 [0.42–0.66], SMD = 0.06). Visual inspection of interaction plots showed similar trends in TDI-MPI and its components after FC in both groups. No significant interactions were identified in any model (all *p* > 0.05) based on linear mixed-effects models, including the time × diastolic dysfunction terms (Supplementary Figures S3, S4).

### Exploratory associations between TDI-MPI normalization after FC and ICU trajectories

**Figure 3:**
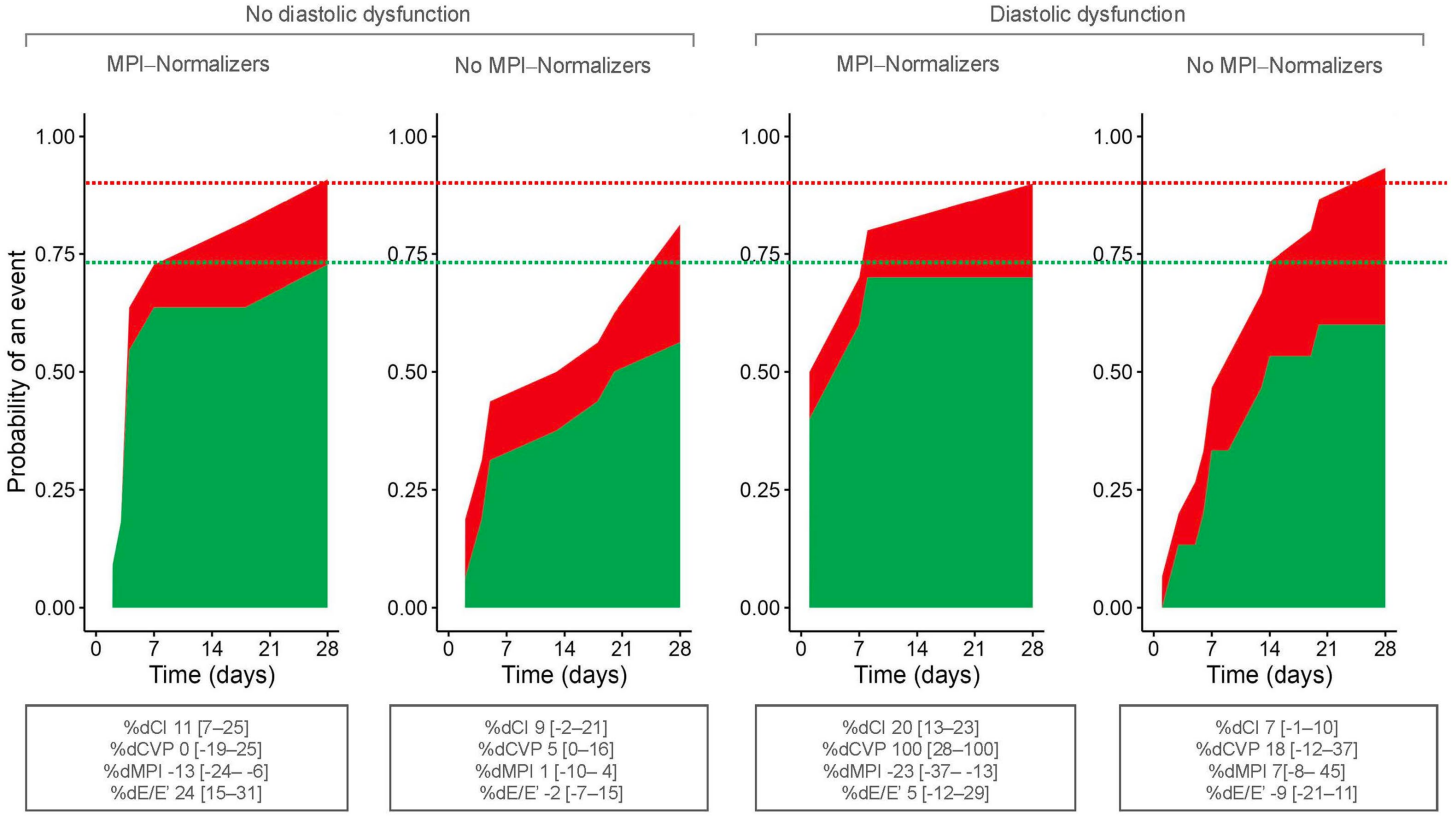
Cumulative incidence of ICU discharge and mortality after fluid challenge by diastolic dysfunction. Cumulative incidence plots showing probabilities of ICU discharge (green) and ICU mortality (red) over 28 days in critically ill patients who received a fluid challenge. Panels are stratified by the presence (right) or absence (left) of diastolic dysfunction and by whether the tissue Doppler imaging-derived myocardial performance index (MPI) normalized after fluid challenge (MPI normalizers vs. non-normalizers). The x-axis indicates time since ICU admission, and the y-axis shows the cumulative probability of ICU discharge or death. Red dashed lines represent cumulative mortality, and green dashed lines indicate cumulative discharge in patients without diastolic dysfunction and MPI normalization. Relative changes (%*Δ*) in cardiac index (CI), CVP, MPI, and the E/E′ ratio are shown below each panel, comparing values before and after fluid challenge.

Stratified visual summaries suggested patterns of higher probabilities of earlier ICU discharge and lower ICU mortality in MPI normalizers, irrespective of baseline diastolic dysfunction (Figure 3). Bayesian survival modeling provided complementary support for these descriptive trends, estimating a 95.2% probability that MPI normalizers experienced a shorter time to ICU discharge (posterior median HR 1.82; 95% CrI, 0.89–3.64).

## Discussion

The main findings of this study can be summarized as follows: (1) TDI-MPI significantly decreased in fluid responders but remained unchanged in non-responders, with MPI improvement after FC being closely associated with fluid responsiveness; (2) the pattern of TDI-MPI change after FC was similar in patients with and without diastolic dysfunction as defined by E/e′ in this study, both among responders and non-responders; and (3) the overall decrease in TDI-MPI after FC in fluid responders was primarily driven by a reduction in IVRT and an increase in ET, whereas IVCT remained largely unchanged.

This study provides an initial evaluation of TDI-MPI changes during FC in critically ill patients. The sample size enabled assessment of TDI-MPI variations across different diastolic function profiles. The prospective design, broad inclusion criteria, and TDI-MPI measurements shortly after ICU admission allowed pragmatic evaluation of this noninvasive parameter through TTE in actual ICU settings. The standardized methodology, which included a predefined FC protocol and measurements performed immediately before and after FC, enhances the validity of our findings. In addition, the use of TDI-MPI, along with minimal changes in heart rate, permitted concurrent assessment of systolic and diastolic phases within a single cardiac cycle, minimizing the beat-to-beat variability commonly observed with pulsed-wave Doppler (27, 28). Collectively, these results support the use of TDI-MPI to refine hemodynamic assessment strategies in critically ill patients undergoing FC.

Non-responders to FC showed no significant changes in TDI-MPI. Although major preload variations can influence TDI-MPI (29–31), previous studies have shown that it remains largely unaffected by preload changes induced by hemodialysis (30–33), increases in positive end-expiratory pressure (PEEP) (34), or alterations in body position (35). In the present study, non-responders provided a clinically relevant model to evaluate TDI-MPI during an acute increase in loading conditions without a concomitant increase in CI. Although FC is expected to increase both mean systemic filling pressure (MSFP) and CVP (36), a significant increase in CVP was observed only in non-responders despite comparable fluid volume and infusion rate between groups. Because mean systemic filling pressure was not measured and CVP was assessed only before and after FC, we could not evaluate the dynamic MSFP–CVP relationship during FC. However, the post-FC increase in CVP supports increased filling pressure/loading conditions in non-responders (37). The stability of TDI-MPI in these patients suggests that this isolated increase in loading conditions was insufficient to alter TDI-MPI. These findings reinforce prior evidence and support the relative preload independence of TDI-MPI in critically ill patients.

Unlike previous studies on preload reduction and TDI-MPI (30–35), our study examined the effects of preload increase. FC augments preload, which may either enhance systolic contraction via the Frank–Starling mechanism or cause LV overload (38). Studies using catecholamines to increase contractility have reported decreased TDI-MPI, primarily due to shortened IVCT and IVRT without changes in ET (29, 39, 40). In contrast, fluid overload can prolong ET, thereby reducing TDI-MPI (14, 29). In the present study, ET increased in both responders and non-responders after FC; however, only responders exhibited a significant decrease in IVRT, whereas IVCT remained largely unchanged. This suggests that FC improved CI in responders primarily through a more favorable left atrial-to-left ventricular pressure gradient, which accelerated mitral valve opening and facilitated early ventricular filling, consistent with an improvement in the conduit function of the left atrium (41, 42). Therefore, this study expands the current understanding of how acute increases in preload affect TDI-MPI in terms of cardiac function dynamics, underscoring its value in evaluating the physiological effects of FC.

The relationship between TDI-MPI and filling pressures remains uncertain and may depend on systolic function (23, 43). Baseline TDI-MPI did not differ between patients with diastolic dysfunction and potentially elevated filling pressures and those without, suggesting limited sensitivity for detecting elevated filling pressures at rest. This finding should be interpreted in the context of an ICU population that included patients with both preserved and reduced EF, although preserved EF was predominant. After FC, however, TDI-MPI responses differed dynamically. Responders with diastolic dysfunction and elevated baseline filling pressures showed a marked decrease in TDI-MPI, accompanied by IVRT shortening, despite the expectation that preload expansion could further increase LV end-diastolic pressure in this subgroup. IVRT shortening may reflect improved relaxation, as suggested by Mahjoub et al. (44), who reported increased E′ after FC in fluid-responsive patients with diastolic dysfunction. Alternatively, IVRT shortening may reflect a more favorable atrioventricular pressure gradient rather than improved relaxation alone. Because LV end-diastolic pressure and direct indices of relaxation were not measured, these mechanisms cannot be directly confirmed. Therefore, dynamic changes in TDI-MPI during FC may reflect left ventricular diastolic adaptation to volume expansion rather than changes in filling pressure alone. Whether these changes reflect improved diastolic function should be evaluated in future studies using a more robust assessment of diastolic function and filling pressures.

The findings of this study enhance the current understanding of the clinical relevance of TDI-MPI in evaluating the effects of FC in critically ill patients. Although TDI-MPI cannot replace CI for FC assessment, it remains valuable when CI measurement is not feasible, given its reduced dependence on probe alignment and ultrasound window quality. In our cohort, valid TDI-MPI measurements were achievable even in patients where CI could not be obtained, highlighting its practical relevance in the ICU. Moreover, changes in TDI-MPI did not always parallel fluid responsiveness as measured by CI; some non-responders showed improvement, whereas some responders showed deterioration. This discordance may reflect the limitations of TDI-MPI; nevertheless, the favorable clinical trajectory seen with post-FC TDI-MPI normalization suggests that TDI-MPI may provide additional insights into the FC physiology beyond CI alone. Therefore, our findings suggest that TDI-MPI may offer a potential alternative method for FC assessment when CI cannot be measured, while also providing complementary information when CI is available. Future research should examine whether combining dynamic TDI-MPI with CI enhances fluid challenge assessment and personalized management, while also providing insights into the potential role of TDI-MPI when CI cannot be measured.

This study has certain limitations. First, no formal power analysis was performed because this was an exploratory secondary analysis. The limited sample size may have reduced the ability to detect subgroup differences or interaction effects, and type II error cannot be excluded for negative findings. Nevertheless, the observed TDI-MPI changes provide preliminary estimates for the design and sample size calculation of future confirmatory studies. Second, this was a single-center study, which may limit external validity because fluid challenge practices, patient case-mix, and echocardiographic expertise may differ across ICUs. Third, only patients with regular rhythm were included, and patients with reduced EF (<50%) were underrepresented despite not being excluded by protocol. Detailed pre-ICU medication history was not systematically collected and may have influenced hemodynamic or echocardiographic responses to FC. In addition, PEEP, ventilation mode, and respiratory system compliance were not included in the analysis, although ventilatory settings were kept unchanged during FC. Therefore, residual confounding cannot be excluded. Fourth, TDI-MPI measurements were obtained exclusively from the lateral mitral annulus, potentially overlooking regional variations (45). In addition, RV function and RV–LV interaction were not systematically assessed, limiting evaluation of their influence on fluid tolerance and TDI-MPI changes during FC. Fifth, fluid responsiveness was assessed using TTE and was not confirmed by thermodilution or pulse contour analysis. Although TTE is non-invasive and reflects routine bedside practice, subtle changes in CI or additional pressure-based hemodynamic information may have been missed (5). Sixth, invasive hemodynamic measurements were not available, which could have provided a more precise evaluation of preload, contractility, and diastolic function. Although CVP is expected to increase physiologically after FC (36), some patients in this cohort, including several non-responders, did not show a clear increase. This may reflect both the physiological and technical limitations of CVP measurement (46, 47), particularly in settings with small changes and low baseline values. Nonetheless, insufficient preload augmentation in these patients cannot be entirely excluded and may partly explain the absence of TDI-MPI changes in non-responders. Notably, a standardized FC (4 mL/kg) was administered via a pump, which was expected to produce only mild and progressive preload increases. A previous study (29) reported that TDI-MPI can be influenced by sudden and substantial changes in preload or afterload. Therefore, the results may not apply to protocols involving rapid fluid administration or unstable conditions with large loading variability. Seventh, although there were no exclusion criteria for severe valvular disease or prosthetic valves, none of the included patients had a history or echocardiographic evidence of severe valvular disease (48), such as severe aortic regurgitation or prosthetic valve disease. Finally, diastolic dysfunction was defined using E/e′ alone, which may not fully capture the complexity of diastolic impairment (24), particularly because some patients may have impaired relaxation with non-elevated or low left atrial pressures.

## Conclusion

In this single-center study of critically ill patients, a 4 mL/kg FC decreased TDI-MPI in fluid responders but not in non-responders. TDI-MPI normalization was associated with fluid responsiveness, supporting its potential role in capturing myocardial adaptation to preload increases induced by FC. Future studies should assess whether combining TDI-MPI with additional echocardiographic indices improves the precision of fluid management strategies in critically ill patients.

## Data Availability

The raw data supporting the conclusions of this article will be made available by the authors, without undue reservation.

## Footnotes

1
https://www.r-project.org
