# Supplementary material for: Fluid challenge and left ventricular myocardial performance index assessed by transthoracic echocardiography with tissue Doppler imaging in critically ill patients with regular rhythm
Source: Front Med (Lausanne). 2026 Jun 24;13:1885708. doi: 10.3389/fmed.2026.1885708 (PMC13341587; doi:10.3389/fmed.2026.1885708)
Supplement: Supplementary file 1 [file Supplementary_file_1.docx]

Fluid challenge and left ventricular myocardial performance index assessed by transthoracic echocardiography with tissue Doppler imaging in critically ill patients with regular rhythm

Mircea Tamas Talpoș^1^*, Halit Ozel^1^, Basil Khaled Mohamed Sallam^1^, Dimitrios Velissaris^2^, Rachid Attou^1^, Charalampos Pierrakos^1^

^1^Department of Intensive Care, Brugmann University Hospital, Université Libre de Bruxelles, Brussels, Belgium

^2^ Department of Internal Medicine, University Hospital of Patras, Patras, Greece

Supplementary Material

**
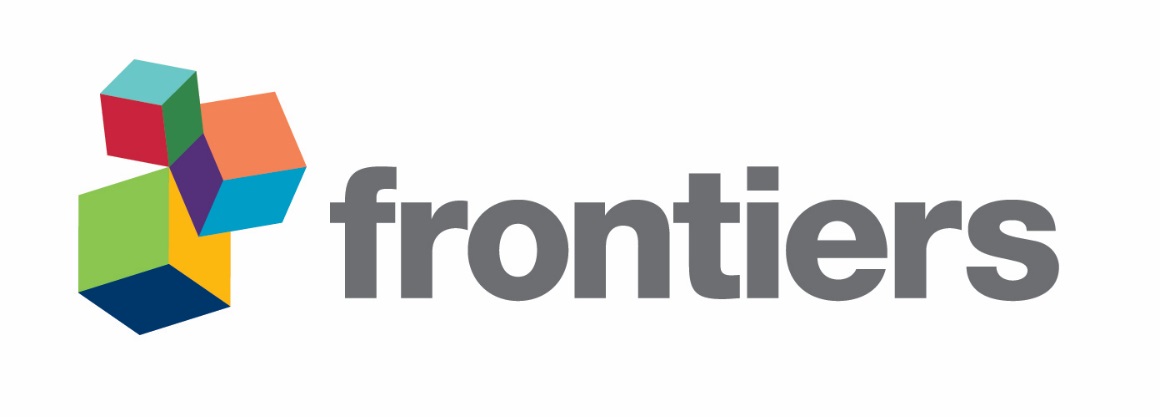
**

**Supplementary Table 1.** H**emodynamic changes before and after fluid challenge according to fluid responsiveness.** Values are presented as median (interquartile range). Comparisons between before and after fluid challenge were performed within each group using the paired Wilcoxon signed-rank test. Fluid responders were defined as patients with an increase in cardiac index ≥15% after fluid challenge.

|  | **Non-responders** | | | **Responders** | | |
| --- | --- | --- | --- | --- | --- | --- |
| **Parameter** | **Before FC** | **After FC** | **p** | **Before FC** | **After FC** | **p** |
| HR | 104 (88–121.5) | 95 (82–113) | 0.001 | 75.5 (64– 86) | 76 (67– 96) | 0.37 |
| SBP | 109 (94–116) | 113 (100–134) | 0.12 | 112 (103–132) | 125 (110–141) | <0.01 |
| DBP | 53 (45–62) | 57 (47–65) | 0.43 | 52 (47–64) | 54 (50–65) | 0.07 |
| MAP | 69.33 (62–79) | 76 (64–89) | 0.11 | 77 (67– 82) | 79 (68–88) | <0.01 |
| PP | 57 (38– 66) | 60 (44–70) | 0.15 | 57 (45– 80) | 70.5 (49–85) | <0.01 |
| SV | 44 (34– 55) | 46 (35–58) | 0.24 | 44 (32–56) | 60 (39–75) | <0.01 |
| CI | 2.18 (1.67–2.79) | 2.27 (1.74–2.86) | 0.82 | 1.91 (1.33–2.33) | 2.27 (1.84–3.23) | <0.01 |
| VTI | 13.2 (11.2–18.3) | 15.8 (12.5–18.7) | 0.31 | 13.6 (11.1–18.4) | 19.4 (13.4–22.1) | <0.01 |
| CVP | 7.5 (2–10.3) | 9.5 (6.2–12.5) | <0.01 | 3.5 (1.8–7.3) | 5.2 (2.1–7.4) | 0.17 |
| **CI**: cardiac index; **CVP**: central venous pressure; **DBP**: diastolic blood pressure; **FC**: fluid challenge; **HR**: heart rate; **MAP**: mean arterial pressure; **PP**: pulse pressure; **SBP**: systolic blood pressure; **SV**: stroke volume; **VTI**: velocity-time integral. | | | | | | |

**Supplementary Table 2**. Patients' hemodynamic and echocardiographic parameters at baseline, before fluid challenge (FC), grouped by their response to FC and the presence or absence of diastolic dysfunction (Diast. Dfc.). Values are expressed as median (interquartile range). Standardized mean differences (SMD) refer to comparisons between the total population of FC responders and non-responders.

|  | **FC responders** | | | **FC non-responders** | | | **SMD** |
| --- | --- | --- | --- | --- | --- | --- | --- |
|  | *Total Population* | *Diast. Dfc.* | *No Diast Dfc* | *Total Population* | *Diast. Dfc.* | *No Diast Dfc* |  |
| No of patients | 24 | 10 | 14 | 35 | 17 | 18 |  |
| **Baseline Hemodynamic parameters** | | | |  | | |  |
| Mean arterial pressure (mmHg) | 77 (67–82) | 71 (57–81) | 79 (72–85) | 69 (62–79) | 68 (58–72) | 76 (65–87) | 0.249 |
| Pulse Pressure (mmHg) | 57 (45–80) | 70 (51–85) | 50 (44–63) | 57 (38–66) | 58 (41–61) | 56 (39–77) | 0.267 |
| Central Venous Pressure (mmHg) | 3 (1–7) | 5 (1–8) | 3 (1–6) | 7 (2–10) | 8 (5–10) | 7 (1–9) | 0.710 |
| Heart rate (beats/min) | 75 (64–86) | 74 (65–78) | 80 (64–116) | 104 (88–121) | 108 (92–122) | 89 (68–120) | 0.606 |
| Stroke Volume (mL) | 44 (32–57) | 49 (37–59) | 38 (31–53) | 44 (34–56) | 49 (31–56) | 44 (35–54) | 0.041 |
| Cardiac Index (L/min/m^2^) | 1.9 (1.3–2.3) | 1.9 (1.3–2.2) | 1.8 (1.3–2.5) | 2.2 (1.7–2.8) | 2.1 (1.6–2.7) | 2.3 (1.9–3.1) | 0.508 |
| **Baseline Echocardiographic parameters** | | | |  | | |  |
| VTI (cm) | 14(11–18) | 16 (13–19) | 12 (11–16) | 13 (11–18) | 13 (12–18) | 14 (11–19) | 0.093 |
| E (cm/sec) | 63 (47–84) | 70 (61–76) | 47 (38–61) | 63 (49–87) | 79 (64–91) | 53 (41–63) | 0.293 |
| A (cm/sec) | 74 (61–87) | 79 (65–83) | 70 (52–88) | 64 (44–81) | 51 (33–70) | 69 (52 –84) | 0.715 |
| E/A | 0.8 (0.7–1.1) | 0.7 (0.3–1.1) | 0.8 (0.4–1.1) | 0.9 (0.7–1.3) | 1.3 (0.8–1.6) | 0.8 (0.5–1.0) | 0.127 |
| E wave deceleration time (ms) | 233 (200–268) | 238 (226–253) | 221 (160–280) | 216 (195–263) | 240 (203–263) | 208 (172–260) | 0.195 |
| S’ (cm/sec) | 9 (7–11) | 8 (7–9) | 10 (8–11) | 9 (7–12) | 7 (6–11) | 9 (7–13) | 0.119 |
| E’ (cm/sec) | 8 (7–10) | 6 (5–8) | 10 (8–12) | 10 (7–11) | 8 (6–9) | 10 (9–12) | 0.265 |
| E/E’ | 8.4 (4.9–13) | 10 (9–12) | 5 (4–6) | 6.8 (5.4–9.2) | 9 (8–11) | 5 (4–6) | 0.102 |
| IVCT (ms) | 58 (44–68) | 53 (45–65) | 61 (47–68) | 52 (48–68) | 56 (50–68) | 52 (44–59) | 0.271 |
| IVRT (ms) | 68 (53–77) | 78 (60–83) | 63 (44–68) | 52 (38–70) | 52 (44–70) | 46 (27–66) | 0.148 |
| Ejection Time (ms) | 263 (177–280) | 269 (256–279) | 248 (156–280) | 196 (161–225) | 200 (175–235) | 194 (158–222) | 0.681 |
| TDI-MPI | 0.48 (0.43–0.58) | 0.46 (0.43–0.49) | 0.51 (0.42–0.61) | 0.49 (0.41–0.81) | 0.49 (0.39–0.76) | 0.48 (0.42–0.74) | 0.306 |
| **A**: Peak late (atrial) diastolic velocity;  **Diast Dfc**: Diastolic Dysfunction; **E**: Peak early (passive) diastolic velocity;  **E’**: early diastolic mitral annular velocity;  **FC**: Fluid Challenge;  **IVCT**: Isovolumic Contraction Time; **IVRT**: Isovolumic Relaxation Time; **S’**: Peak systolic velocity of the mitral annulus; **SV**: Stroke Volume; **TDI-MPI**: Tissue Doppler Imaging Myocardial Performance Index; **VTI**: Velocity-Time Integral. | | | | | | | |

**Supplementary Table 3**. Relative (%) and absolute (Δ) changes of hemodynamic and echocardiographic parameters after fluid challenge (FC). The patients were grouped by their response to FC and the presence or absence of diastolic dysfunction (Diast. Dfc.). Values are expressed as median (interquartile range). Standardized mean differences (SMD) refer to comparisons between the total population of FC responders and non-responders.

|  | **FC responders** | | | **FC non-responders** | | | **SMD** |
| --- | --- | --- | --- | --- | --- | --- | --- |
|  | *Total Population* | *Diast. Dfc.* | *No Diast Dfc* | *Total Population* | *Diast. Dfc.* | *No Diast Dfc* |  |
| No of patients | 24 | 10 | 14 | 35 | 17 | 18 |  |
| **Hemodynamic parameters changes during FC** | | | |  | | |  |
| delta mean arterial pressure (%) | 5 (2–7) | 4 (-2–5) | 5 (3–7) | 4 (-4–11) | -1 (-4–8) | 4 (-4–12) | 0.289 |
| Δ mean arterial pressure (mmHg) | 3 (2–5) | 2 (-2–4) | 4 (2–5) | 2 (-3–8) | 3 (-4–10) | -1 (-3–5) | 0.217 |
| delta pulse pressure (%) | 12 (7–16) | 12 (8–14) | 13 (6–19) | 4 (-10–26) | 2 (-7–13) | 5 (-10–27) | 0.042 |
| Δ pulse pressure (mmHg) | 8 (5–11) | 7 (5–8) | 8 (4–12) | 2(-5–14) | 1 (-3–7) | 3 (-5–17) | 0.145 |
| delta Central Venous Pressure (%) | 0 (-33–30) | 37 (30–100) | -33 (-53–0) | 17 (0–44) | 18 (1–33) | 16 (0–44) | 0.465 |
| Δ Central Venous Pressure (mmHg) | 2 (0–3) | 2 (1–3) | 0 (-1–1) | 1 (-0.3–3) | 2 (0–4) | 1 (0–2) | 0.445 |
| delta Heart rate (%) | -1 (-5–3) | -2 (-5–4) | 1 (-5–3) | -4 (-9–1) | -4 (-10– -2) | -1 (-7–2) | 0.434 |
| Δ Heart rate (beats/min) | -1 (-5–2) | -2 (-5–3) | 1 (-4–2) | -2 (-10–1) | -4 (11– -1) | -1 (-9–2) | 0.567 |
| delta Stroke Volume (%) | 28 (19–38) | 26 (18–48) | 28 (22–36) | 5 (-5–13) | 5 (-6–15) | -2 (-4–12) | 1.577 |
| Δ Stroke Volume (mL) | 12 (8–21) | 12 (10–21) | 11 (7–21) | 2 (-3–6) | 3 (-4–7) | 1 (-2–5) | 1.776 |
| delta Cardiac Index (%) | 25 (20–32) | 23 (20–42) | 28 (19–31) | 1 (-5–8) | 1 (-7–7) | 1 (-3–8) | 1.073 |
| Δ Cardiac Index (L/min/m^2^) | 0.5 (0.4–0.6) | 0.5 (0.4–0.6) | 0.6 (0.4–0.6) | 0.1 (-0.2–0.16) | 0.0 (-0.1–0.2) | 0.0 (-0.2–0.2) | 1.877 |
| **Echocardiographic parameters changes during FC** | | | |  | | |  |
| delta VTI (%) | 28 (18–37) | 26 (17–48) | 27 (21–36) | 5 (-5–13) | 8 (-6–18) | 1 (-4–10) | 1.445 |
| Δ VTI (cm) | 4.1 (2.5–6.1) | 3.8 (2.3–5.9) | 4.2 (3.4–6.6) | 0.6 (-1.1–1.8) | 0.9 (-1.4–2.2) | 0.1 (-0.7–1.5) | 1.675 |
| delta E (%) | 13 (5–29) | 11 (5–12) | 22 (7–37) | 6 (-6–17) | 2 (-11–7) | 7 (-4–27) | 0.480 |
| Δ E (cm/sec) | 9 (3–17) | 7 (4–9) | 9 (4–18) | 4 (-5–10) | 2 (-7–7) | 5 (-1–12) | 0.664 |
| delta A (%) | 7 (-2–15) | 11 (0–19) | 4 (-3–16) | -3 (-9–8) | 0 (-24–5) | -3 (-8–10) | 0.433 |
| Δ A (cm/sec) | 4 (0–19) | 7 (0–15) | 1 (-1–23) | 3 (-8–3) | 0 (-8–1) | -4 (-7–5) | 0.565 |
| delta E/A (%) | 15 (-8–33) | 7 (-15–24) | 22 (1–37) | 14 (-1–39) | 9 (-4–27) | 20 (0–40) | 0.185 |
| Δ E/A | 0.13 (-0.01–0.28) | 0.01 (-0.01 –12) | 0.27 (0.05–0.29) | 0.06 (-0.01–0.23) | 0.04 (-0.02–0.15) | 0.16 (0.00–0.62) | 0.218 |
| delta E wave Desceleartion time (%) | -9 (-21–0) | -9 (-20–5) | -8 (-27– -5) | -7 (-18–0) | -6 (-15–22) | -9 (-17– -2) | 0.279 |
| Δ Desceleartion Time (ms) | -19 (-66–0) | -23 (-53–12) | -19 (-66– -13) | -15 (-46–2) | -13 (-40– 30) | -21 (-45– -3) | 0.225 |
| delta S’ (%) | 0.6 (-6–11) | 2.1 (-1.9–11.1) | 0.6 (-11–9) | 4 (-19–16) | 8 (-15–12) | -1 (-22–15) | 0.061 |
| Δ S’ (cm/sec) | 0.05 (-0.55–1.05) | 0.15 (-0.01–1.0) | 0.05 (-1.21–1.15) | 0.6 (-1.9–1.12) | 0.8 (-1.6–1) | 0.15 (-2.3–1) | 0.139 |
| delta E’ (%) | 13 (0–24) | 8 (0–13) | 22 (4–26) | 0 (-11–14) | 0 (-10–10) | 11 (-16–20) | 0.088 |
| Δ E’ (cm/sec) | 1 (0–2) | 1.0 (0.0–1.6) | 1.5 (0.1–2.0) | 0 (-1–1) | 0.0 (-1.0 –1.0) | 0.7 (-1.5–1.3) | 0.284 |
| delta E/E’ | 11 (-8–30) | 14 (1–35) | 1 (-17–18) | 4 (-11–19) | 8 (-5–23) | -5 (-16–13) | 0.253 |
| Δ E/E’ | 0.9 (-0.6–1.6) | 0.9 (0.1–1.6) | 0.1 (-1.9–1.7) | 0.1 (-0.6–1.1) | 0.5 (-0.2–1.2) | -0.6 (-1.8–1.1) | 0.270 |
| delta IVCT (%) | 0 (-6–28) | 0 (-5–7) | -7 (-5–38) | -6 (22–14) | -9 (-22–15) | 6 (-23–13) | 0.513 |
| Δ IVCT (ms) | 0 (-4–15) | 0 (-3–3) | -3 (-7–10) | -4(-11–8) | -4 (-9–8) | 2 (-15–7) | 0.567 |
| delta IVRT (%) | -17 (-36–7) | -23 (-45–8) | -11 (31 –6) | 0 (-18–27) | 3 (-16–29) | 0 (-18–27) | 0.513 |
| Δ IVRT (ms) | -8 (-28–4) | -15 (-37–6) | -6 (-18–4) | 0 (-10–10) | 1 (-18–10) | 0 (-17–9) | 0.298 |
| delta Ejection Time (%) | 4 (-1–12) | 5 (1–11) | 3 (-2–11) | 6 (0–19) | 8 (1–17) | 3 (-1–14) | 0.306 |
| Δ Ejection Time (ms) | 8 (-4–28) | 8 (2–27) | 7 (-6–30) | 13 (0–37) | 16 (2–38) | 8 (-3–27) | 0.091 |
| delta TDI-MPI (%) | -11 (-27–8) | -23 (-37–8) | -10 ( -11–6) | -3 (-21–5) | 2 (-24–30) | -4 (-19–1) | 0.08 |
| Δ TDI-MPI | -0.05 (-0.14–0.03) | -0.11 (-0.17–0.03) | -0.04 (-0.07–0.02) | -0.01 (-0.11–0.02) | 0.01 (- 0.02–0.01) | -0.02 (- 0.08–0.01) | 0.401 |
| **A**: Peak late (atrial) diastolic velocity;  **Diast Dfc**: Diastolic Dysfunction; **E**: Peak early (passive) diastolic velocity;  **E’**: early diastolic mitral annular velocity;  **FC**: Fluid Challenge;  **IVCT**: Isovolumic Contraction Time; **IVRT**: Isovolumic Relaxation Time; **S’**: Peak systolic velocity of the mitral annulus; **SV**: Stroke Volume; **TDI-MPI**: Tissue Doppler Imaging Myocardial Performance Index; **VTI**: Velocity-Time Integral. | | | | | | |  |

**Supplementary Figure 1.** Tissue Doppler Imaging of the lateral mitral annulus combined with electrocardiographic readings. The isovolumetric contraction time (IVCT), isovolumetric relaxation time (IVRT), and ET were obtained from the TDI trace. TDI-MPI was calculated as the ratio of the sum of the IVCT and IVRT to ET.


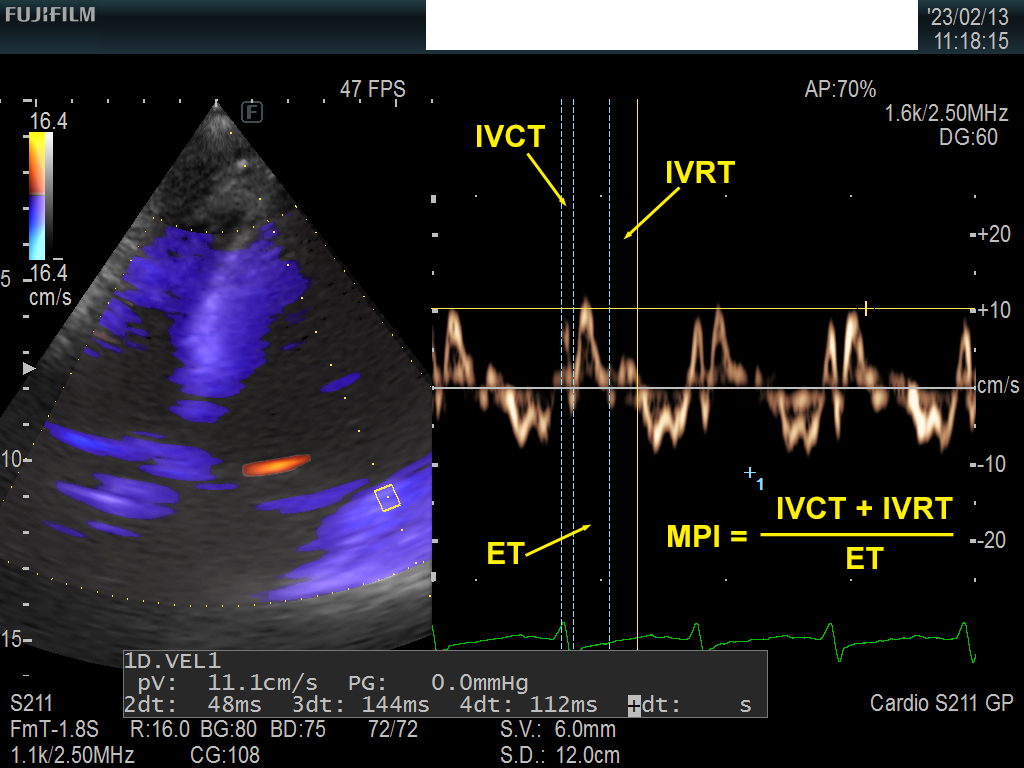


**Supplementary Figure 2.** Flowchart of patient selection. Patients with P_va_CO_2_ <6 mmHg were not included in the primary study cohort but were eligible for this analysis if complete TDI-MPI and CI data were available.


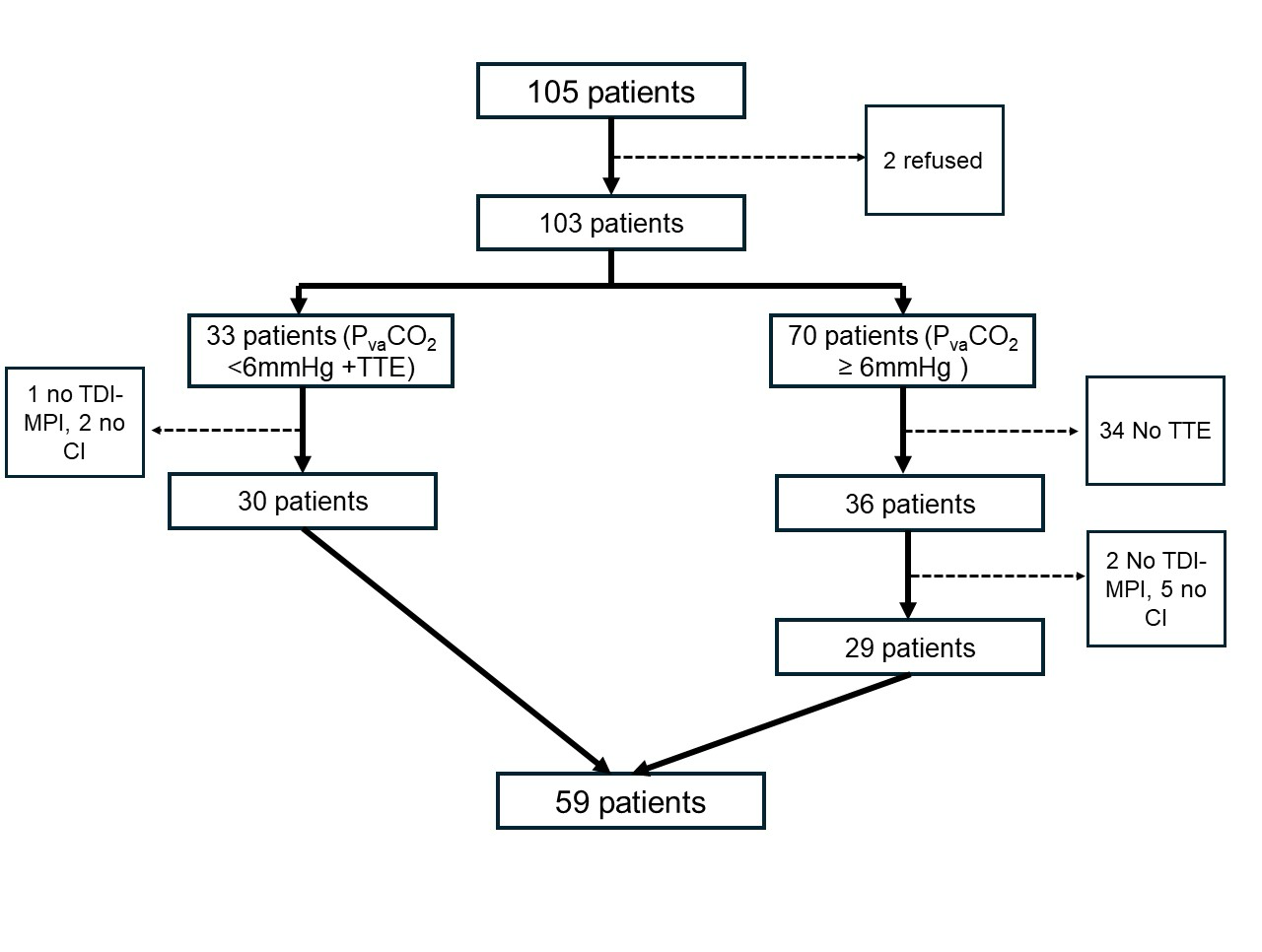


**Supplementary Figure 3.**Interaction plot showing tissue Doppler imaging myocardial performance index (TDI–MPI) values before and after fluid challenge (FC) in fluid responders and non-responders, stratified by diastolic function status. The left panel displays TDI–MPI changes in non-responders, and the right panel shows TDI–MPI changes in responders. Blue lines represent patients without diastolic dysfunction, and red lines represent patients with diastolic dysfunction.


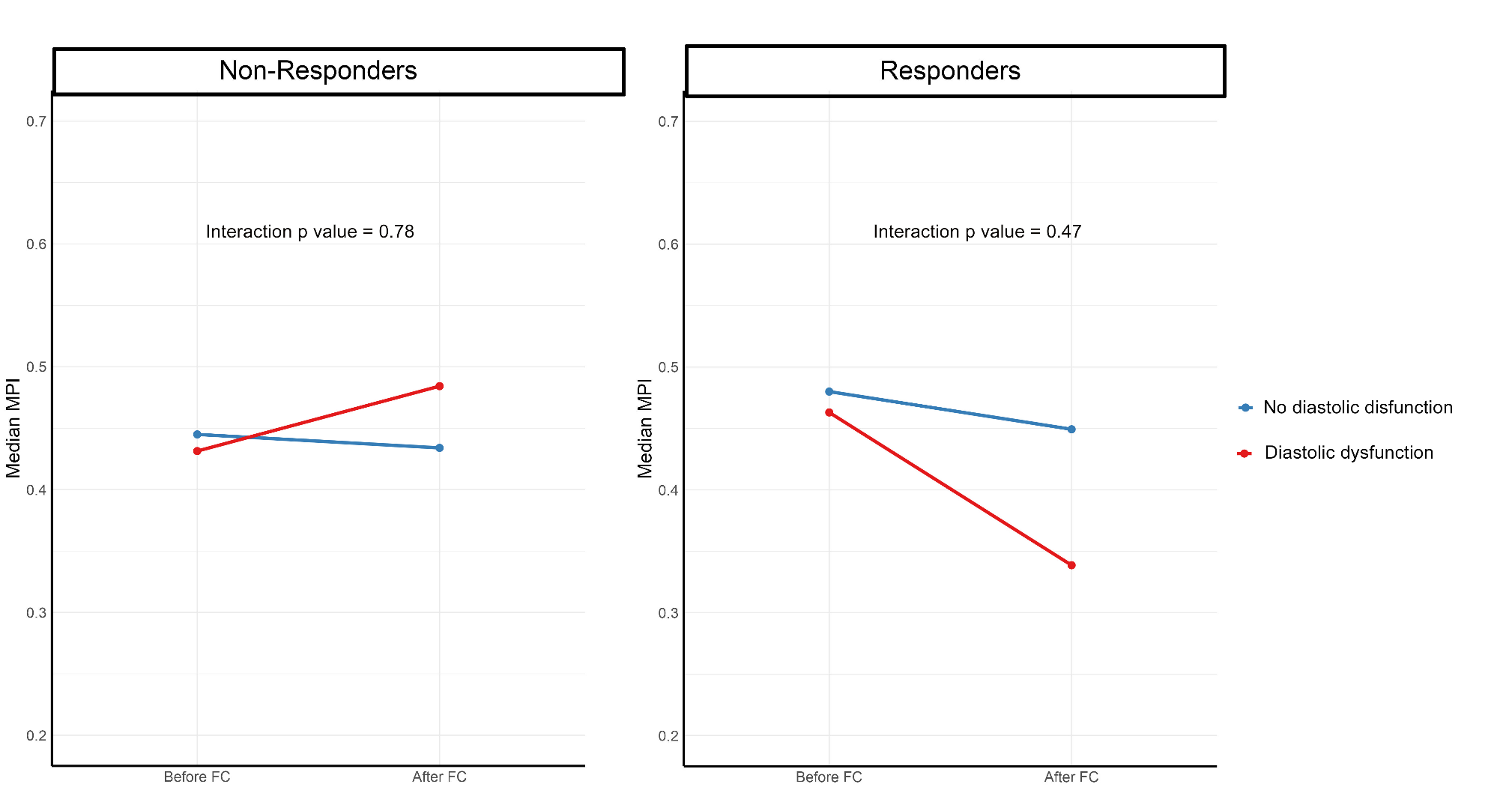


**Supplementary Figure 4.** Interaction plot showing median values components of the tissue Doppler imaging myocardial performance index (TDI-MPI) before and after fluid challenge (FC) in fluid responders and non-responders, , stratified by diastolic function status. Each panel illustrates a different component: the left panel isovolumic contraction time (IVCT), the middle panel shows isovolumic relaxation time (IVRT), and the right panel shows ejection time (ET). Blue lines represent patients without diastolic dysfunction, and red lines represent patients with diastolic dysfunction.


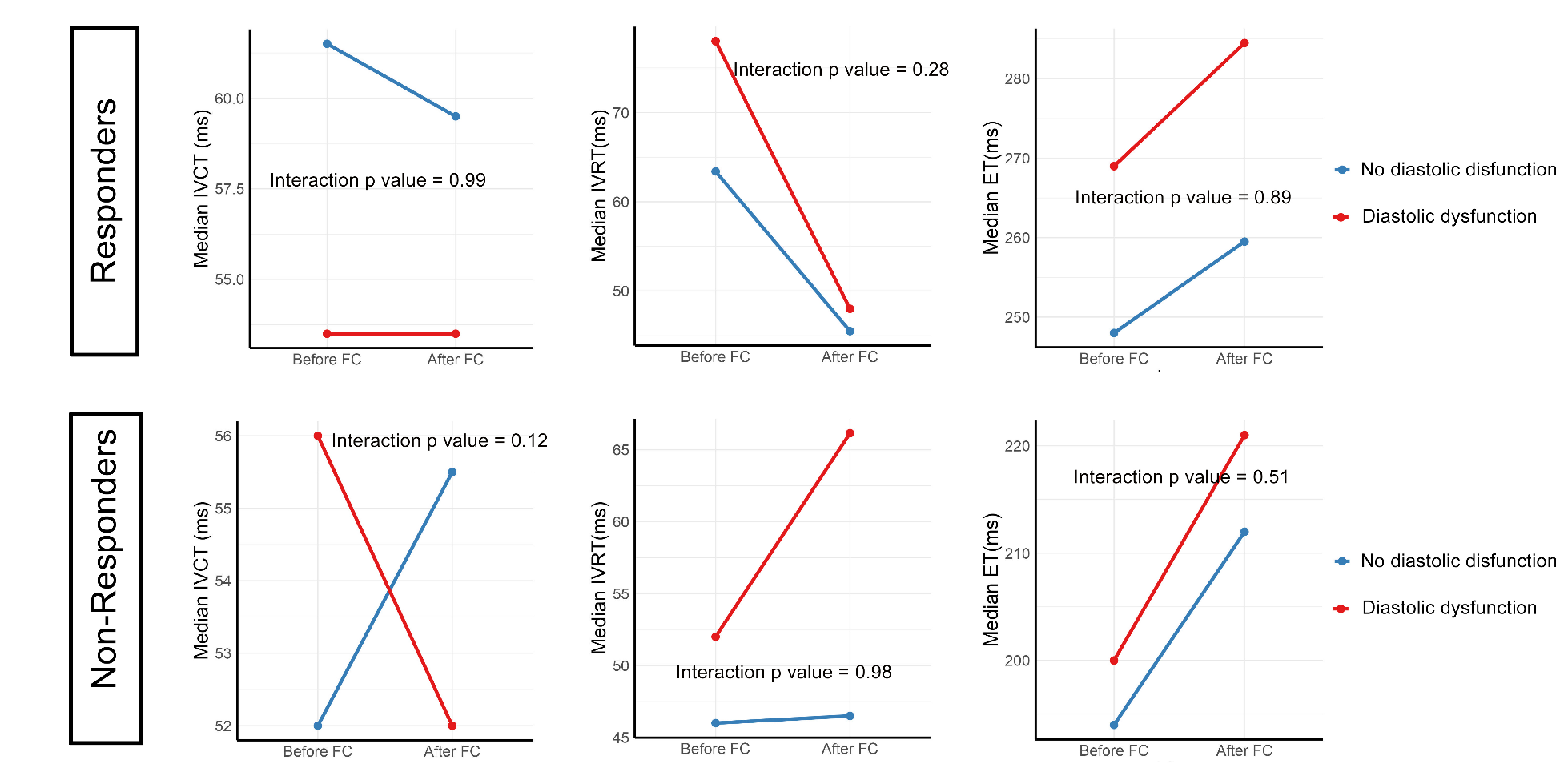


**Supplementary Figure 5.** Logistic regression analysis examining the association between fluid challenge (FC) responsiveness tissue Doppler imaging myocardial performance index (TDI-MPI) normalization after FC. The analysis was performed on the total population and stratified by diastolic function status (Not-Diastolic Dysfunction and Diastolic Dysfunction). Odds ratios (OR) and 95% confidence intervals (CI) are displayed, with the x-axis representing the odds ratio (95% CI) for fluid responsiveness associated with TDI-MPI normalization.

**
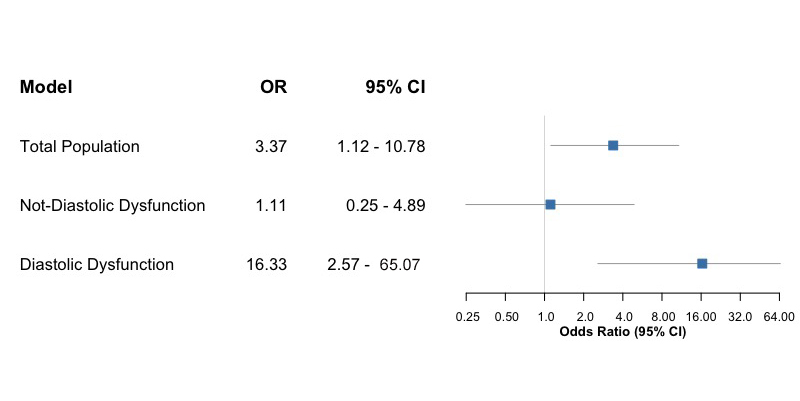
**
